# Strain-level diversity in sulfonamide biodegradation: adaptation of *Paenarthrobacter* to sulfonamides

**DOI:** 10.1093/ismejo/wrad040

**Published:** 2024-01-10

**Authors:** Yue Huang, Anxin Pan, Ying Song, Yu Deng, Alnwick Long-Hei Wu, Colin Shiu-Hay Lau, Tong Zhang

**Affiliations:** Environmental Microbiome Engineering and Biotechnology Lab, Department of Civil Engineering, The University of Hong Kong, Pokfulam Road, Hong Kong SAR 999077, China; Environmental Microbiome Engineering and Biotechnology Lab, Department of Civil Engineering, The University of Hong Kong, Pokfulam Road, Hong Kong SAR 999077, China; Environmental Microbiome Engineering and Biotechnology Lab, Department of Civil Engineering, The University of Hong Kong, Pokfulam Road, Hong Kong SAR 999077, China; Environmental Microbiome Engineering and Biotechnology Lab, Department of Civil Engineering, The University of Hong Kong, Pokfulam Road, Hong Kong SAR 999077, China; Environmental Microbiome Engineering and Biotechnology Lab, Department of Civil Engineering, The University of Hong Kong, Pokfulam Road, Hong Kong SAR 999077, China; Environmental Microbiome Engineering and Biotechnology Lab, Department of Civil Engineering, The University of Hong Kong, Pokfulam Road, Hong Kong SAR 999077, China; Environmental Microbiome Engineering and Biotechnology Lab, Department of Civil Engineering, The University of Hong Kong, Pokfulam Road, Hong Kong SAR 999077, China

**Keywords:** sulfonamide, biodegradation, Paenarthrobacter, *sad* genes, transposable element

## Abstract

The widespread occurrence of sulfonamides raises significant concerns about the evolution and spread of antibiotic resistance genes. Biodegradation represents not only a resistance mechanism but also a clean-up strategy. Meanwhile, dynamic and diverse environments could influence the cellular function of individual sulfonamide-degrading strains. Here, we present *Paenarthrobacter* from different origins that demonstrated diverse growth patterns and sulfonamide-degrading abilities. Generally, the degradation performance was largely associated with the number of *sadA* gene copies and also relied on its genotype. Based on the survey of *sad* genes in the public database, an independent mobilization of transposon-borne genes between chromosome and plasmid was observed. Insertions of multiple *sadA* genes could greatly enhance sulfonamide-degrading performance. Moreover, the *sad* gene cluster and *sadA* transposable element showed phylogenetic conservation currently, being identified only in two genera of *Paenarthrobacter* (*Micrococcaceae*) and *Microbacterium* (*Microbacteriaceae*). Meanwhile, *Paenarthrobacter* exhibited a high capacity for genome editing to adapt to the specific environmental niche, opening up new opportunities for bioremediation applications.

## Graphical Abstract

**Graphical Abstract:**
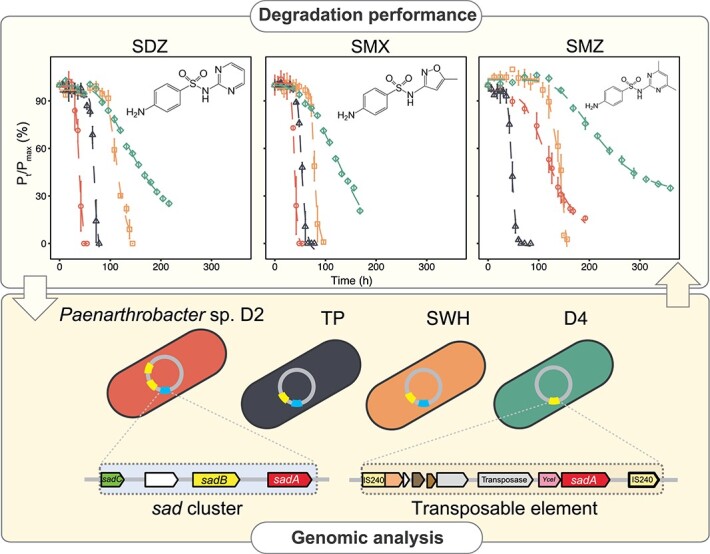

## Introduction

Sulfonamides are among the most widely used antibiotics for both human and veterinary applications chiefly because of their broad inhibition ability for bacteria [1, 2]. However, there are growing concerns regarding their widespread distribution in various natural environments. In the past decades, sulfonamides have been frequently detected in wastewater [3, 4], surface water [5, 6], groundwater [6, 7], and drinking water [8, 9] around the world in concentrations from nanograms to micrograms per liter. In particular, sulfamethoxazole, sulfadiazine, and sulfamethazine have been observed with the highest reported human consumption and detection rates in aquatic environments [10-12]. The residual sulfonamide can accelerate the evolution and spread of antibiotic resistance genes [13], posing a great threat to ecosystems and global health.

Although the evolution of bacteria to gain antibiotic resistance has long been appreciated, our knowledge of the involved mechanisms has increased significantly in recent years. Well-documented antibiotic resistance mechanisms include prevention of access to the target (e.g. efflux pumps), changes in structure and modification of antibiotic targets (e.g. mutation), and direct modification of antibiotics (e.g. hydrolysis) [14]. In addition, antibiotic subsistence in bacteria is an alternative resistance mechanism, while importantly, it can inactivate antibiotics and reduce environmental concentrations, representing a cure for environmental resistance. In the past two decades, a wide variety of sulfonamide-catabolizing bacterial strains have been isolated, spanning diverse bacterial genera [15, 16]. Nevertheless, the *sad* gene cluster is the sole experimentally validated sulfonamide-degrading gene cluster so far [17, 18]. In *Microbacterium* spp. BR1 and CJ77, e.g. sulfonamides were initially attacked by the flavin-dependent monooxygenase encoded by the *sadA* gene (the homologous gene *sulX* in CJ77) with the generation of 4-aminophenol, the corresponding dead-end metabolites, and sulfite (Fig. S1). Subsequently, another flavin-dependent monooxygenase encoded by the *sadB* gene was responsible for the conversion of 4-aminophenol into 1,2,4-trihydroxybenzene. In this biotransformation pathway, flavin reductase encoded by the *sadC* (*sulR*) gene plays an auxiliary role in electron transport [17, 18]. The complete sulfonamide mineralization was then achieved by the interspecific interactions among sulfonamide degraders and other species, such as *Pimelobacter* [19] and *Acidovorax* [20]. Unlike the phylogenetical diversity of sulfonamide-catabolizing strains, the *sad* genes were only reported in a few sulfonamide degraders affiliated with the families of *Microbacteriaceae* and *Micrococcaceae*. The underlying propagation pattern of sulfonamide-degrading genes associated with the limited spread beyond the boundary of *Microbacteriaceae* and *Micrococcaceae* lineages remains to be elucidated.

Likewise, environmental niches not only shape the structure of microbial communities but also strain-level diversities, resulting in distinct functional performance and influencing intraspecific or interspecific interactions [21]. However, strain-level variation is frequently overlooked in surveys of community structure due to the limitations inherent in marker gene-based analysis. A finer-grained assessment of genetic diversity is heavily dependent on technological revolution. Recent advances in sequencing technologies have allowed microbiologists to determine the functions of individual strains according to the circular genome instead of just genetic fragments [22, 23]. Despite the fact that the strain-level differentiation of pollutant-degrading capacities among a single genus is of vital importance and significance in practical applications, relevant studies are very limited.

Our study focused on *Paenarthrobacter*, a bacterial genus first described in 2016 and previously known as *Arthrobacter* [24], which is the genus most of the reported sulfonamide degraders were affiliated with. We hypothesized that *Paenarthrobacter* would exhibit functional diversity despite the high genetic similarity of individual strains. In this study, we presented the genomic characteristics and growth kinetics of eight *Paenarthrobacter* strains isolated from sulfonamide-degrading enrichments seeded with activated sludge from different sewage treatment plants. Then, we conducted a phylogenetic analysis of the *sad* genes and degradation experiments to reveal the sulfonamide-degrading capability of four selected strains with different *sad* arrangements. Furthermore, a survey of *sad* genes in the public database was performed to investigate potential degraders and propagation patterns of sulfonamide-degrading genes.

## Materials and methods

### Chemicals and pure strains

Sulfadiazine (SDZ), sulfamethoxazole (SMX), sulfamethazine (SMZ), and formic acid were purchased from Sigma-Aldrich (USA). Liquid chromatography/mass spectrometry–grade acetonitrile was purchased from Fisher Chemicals (Pittsburgh, PA). Ultrapure water was produced by a Barnstead EASYpure UV/UF water purification system. As described in our previous studies [25, 26], the *Paenarthrobacter* spp. were isolated from eight SDZ-degrading enrichments seeded with different activated sludge from six local sewage treatment works.

### Growth kinetics

*Paenarthrobacter* spp. used in this study were kept frozen at −80°C in Luria-Bertani (LB) medium with 20% (v/v) glycerol. Before the experiment, a 50-μl frozen suspension was inoculated into 30 ml of liquid LB medium and incubated at room temperature for 24 hours. The *Paenarthrobacter* sp. was purified by streaking on LB agar plates (1.5% agar). Then, a single colony was used to inoculate 30 ml of LB medium and incubate for ~48 hours (log phase based on observation). In the next step, the dense culture was inoculated in a ratio of 1:100 into a fresh 30 ml of LB medium in triplicate. Cell density was measured by optical density at 595 nm (OD_595_) with an iMark Microplate Absorbance Reader (Bio-Rad, Hercules, CA, USA). The logistic model was used to describe the growth pattern. The growth kinetics were determined in LB medium without sulfonamide (pre-antibiotic). In addition, the post-antibiotic replication rates of the isolates under sulfonamide pressure (100 mg/L SDZ) were estimated using the growth rate index (GRiD, v1.3) [27] in single mode.

### Sulfonamide degradation experiment

Four *Paenarthrobacter* spp. were selected for degradation experiments based on their different *sad* gene arrangements. The *Paenarthrobacter* spp. were grown in fresh LB medium at room temperature with shaking at 180 rpm to exponential phase (OD_595_ of 1.0–1.5). The biomass was harvested by centrifugation at 3725 *g* for 20 min (Beckman Coulter Avant J-15R) and washed with mineral salt medium (MSM, Table S1) trice. The biodegradation experiment was carried out in triplicate in 250-ml Erlenmeyer flasks containing 150 ml of MSM amended with 100 mg/L sulfonamide as the sole carbon source. Inoculum of *Paenarthrobacter* sp. was added in a set of three flasks to a target initial OD_595_ of 0.1. The control treatment was inoculated with mixed sterilized cultures to monitor the abiotic loss of sulfonamides. All the flasks were sealed with sterile breathable sealing film and incubated at room temperature and 180 rpm. A 2-ml suspension of *Paenarthrobacter* sp. was withdrawn at designed intervals from three independent flasks and centrifuged at 20000 *g* for 2 min in a 4°C pre-chilled centrifuge. The supernatant was filtered with 0.22-μm polyvinylidene fluoride syringe filters (Millipore, Germany) and stored at 4°C until analysis. The modified Gompertz model was applied to fit the degradation data.

### Sequencing and assembly of *Paenarthrobacter* spp.

To obtain the circular chromosome of our *Paenarthrobacter* spp., additional Nanopore sequencing for each isolate was performed. Briefly, the DNA of each isolate was extracted using a DNeasy PowerSoil Kit (Qiagen, Germany) following the manufacturer’s instructions. DNA purification was performed with a standard AMPure XP bead (Beckman Coulter) clean-up purification protocol. An equal amount of DNA from each isolate was used for library preparation with a ligation sequencing kit (SQK-LSK109) and purified following the standard AMPure XP bead clean-up protocol. Subsequently, the prepared library was loaded onto an R9 flow cell (FLO-MIN106) for sequencing on a GridION using MinKNOW (v21.10.8). Base calling was processed using Guppy (v5.0.17, Oxford Nanopore Technologies), and raw long reads were processed with Porechop (v0.2.4) (https://github.com/rrwick/Porechop) to remove adapter barcode sequences. The total additional sequencing amount is 14.09 Gb. Together with our previously sequenced Illumina short reads and Nanopore long reads (PRJNA669352), the genome of eight *Paenarthrobacter* spp. was hybrid assembled using Unicycler (v0.5.0) [28]. Whole-genome pairwise average nucleotide identity (ANI) and average amino acid identity (AAI) were calculated using fastANI (v1.32) [29] and CompareM (v0.0.23) AAI workflow (https://github.com/dparks1134/CompareM), respectively. The plasmids were determined by PlasFlow (v1.1) [30] with a 0.8 threshold. Phylogenetic tree construction and taxonomic reassignment were performed by GTDB-tk (v2.1.1) [31] based on Genome Taxonomy Database taxonomy R214. The phylogenetic tree was midpoint rooted and visualized in iTOL (v6) [32]. Subsequently, the open reading frames (ORFs) were predicted by Prodigal (v2.6.3) [33] and annotated by eggNOG-mapper (v2.1.9) [34] against the eggNOG v5.0 database.

### *sad* gene identification and database mining

The GenBank flat files (.gbff) of 433 218 bacteria were downloaded from the NCBI database (updated in July 2023). Then, the nucleotide sequences were extracted by an in-house Python script, and the ORFs were predicted by Prodigal (v2.6.3) [33]. The *sad* genes were identified using DIAMOND (v2.0.8.147) [35] with strict criteria (>70% identity, >70% query length coverage, and <1e-5 e-value). Gene arrangement of sulfonamide-degrading gene clusters was visualized using the R package gggenes (v0.5.0) (https://github.com/wilkox/gggenes).

### Analytical methods

The concentrations of sulfonamides were determined by ultraperformance liquid chromatography–tandem mass spectrometry (Acquity UPLC system, Waters) with positive ion mode ESI. In this method, the extract was gradient eluted from a BEH C18 column (2.1 × 50 mm, 1.7 μm, held at 50°C) using water (A) and acetonitrile (B), containing 0.1% formic acid. The gradient was initially 95% A for 2 min, linearly increased to 78% A at 6.5 min, and returned to 95% A at 6.6 min, with a flow rate of 0.4 ml/min. The sample injection volume was 10 μl. The desolvation and source temperatures were 400 and 120°C, respectively. The desolvation and cone gas (nitrogen) flow rates were 600 and 50 L/h, respectively. Argon was used as the collision gas at a flow rate of 0.15 ml/min. Instrument control and data acquisition were processed with MassLynx software (v4.2, Waters). Multiple reaction monitoring mode was used to monitor the *m/z* transition of sulfonamides and their major metabolites (Table S2).

## Results and discussion

### Taxonomy reassignment of eight isolates

Eight strains used in the present study were isolated from sulfonamide-degrading consortia fed with sulfadiazine as the sole carbon source (100 mg/L). They were formerly identified as *Arthrobacter* spp. In this study, based on additional sequencing data, four complete genomes (D2, D4, SK, and SL) and four draft genomes (SWH, ST, YL, and TP) were achieved with a genome size of 4.78 ± 0.09 Mb and a high average GC content of 63.4%. Each genome comprises a circular chromosome (4.58 ± 0.04 Mb) and multiple plasmids (Table S3). Consistent with the average number of 16S rRNA copies in the genus *Paenarthrobacter* [36], the eight isolates under study contain six copies of the 16S rRNA gene. In a genome-based phylogeny (Fig. S2), all eight isolates were placed together with *Paenarthrobacter ureafaciens* DSM 20126 as a monophyletic sister group within the clade *Paenarthrobacter*, a novel genus proposed in 2016 [24], implying that these strains were properly affiliated with *Paenarthrobacter*. Meanwhile, the isolates were similar to each other, with extremely high ANI and AAI values above 99.9% (Table S4), stimulating interest in investigating the underlying genetic impact on sulfonamide biodegradation at the strain level.

### Phylogeny of *sad* genes

**Figure 1:**
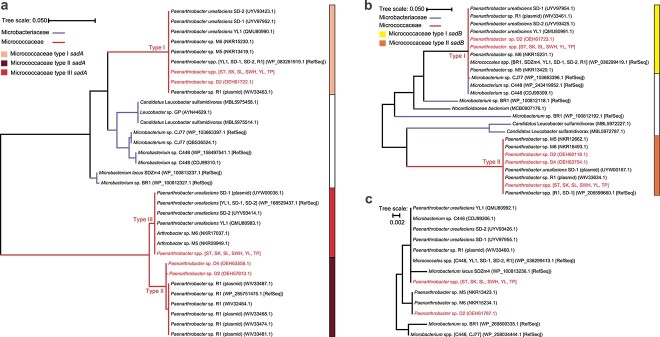
Maximum likelihood trees of *sadA* (A), *sadB* (B), and *sadC* (C) based on amino acid sequences. The strains under study are highlighted.

To date, *sad* gene diversity as well as different arrangements in identified sulfonamide degraders have been reported in a few studies [18, 37]. Here, the recovered *sad* genes together with reference sequences from the NCBI GenBank and RefSeq databases were used to construct the phylogenetic trees. As expected, *sadA* genes were only identified in *Microbacteriaceae* and *Micrococcaceae*. All the *sadA* genes retrieved from our isolates fell into *Micrococcaceae sadA*, being grouped with *sadA* sequences from other *Paenarthrobacter* spp. (Fig. 1A). These *sadA* genes could be further assigned into three clusters based on the topological structure. Specifically, *sadA* Type II shared 97.5% identity with Type III, whereas *sadA* Type I was homologous to Types II and III with 78.9% and 79.2% amino acid identities, respectively. Among the eight isolates, three different arrangements of *sadA* were found, namely, (i) Type I *sadA* and Type II *sadA* (*Paenarthrobacter* sp. D2), (ii) Type I *sadA* and Type III *sadA* (*Paenarthrobacter* spp. ST, SK, SL, SWH, YL, TP), and (iii) Type II *sadA* only (*Paenarthrobacter* sp. D4).

In comparison, the diversity of *sadB* and *sadC* retrieved from our isolates was relatively lower. Analogous to *sadA* genes, *sadB* genes could be divided into *Micrococcaceae sadB* and *Microbacteriaceae sadB* according to their affiliated families. On the contrary, *Micrococcaceae* Type I *sadB*, adjacent to Type I *sadA*, displayed a high similarity with some *Microbacteriaceae sadB* genes, indicating that they were orthologous genes and originated from a common ancestor. Furthermore, *Micrococcaceae* Type II *sadB* was a plasmid-borne gene, only identified in *Paenarthrobacter* spp., sharing 73.2% AAI with Type I *sadB*. Nevertheless, the expression of the Type II *sadB* during sulfonamide biodegradation remains unclear as it is located remotely from the *sad* gene cluster. In addition to *sadA*-carrying strains, a *sadB* homogenous gene was also identified in a *Nocardioidaceae* bacterium (85.0% identity with Type I *sadB*), which was a metagenome-assembled genome (MAG) reconstructed from the activated sludge sample of the Sha Tin sewage treatment works [38]. The monooxygenase encoded by *sadB* could be involved in the conversion of 4-aminophenol, which entered the municipal wastewater by inevitable release from chemical processes or industries, or as a biotransformation intermediate of dyes and pharmaceuticals [39]. Besides, *sadC* is an accessory gene for sulfonamide biodegradation, which is highly conserved (>99.5% amino acid identity) and located near *sadB* in all experimentally verified degraders.

### Growth kinetics of eight *Paenarthrobacter* spp.

**Figure 2:**
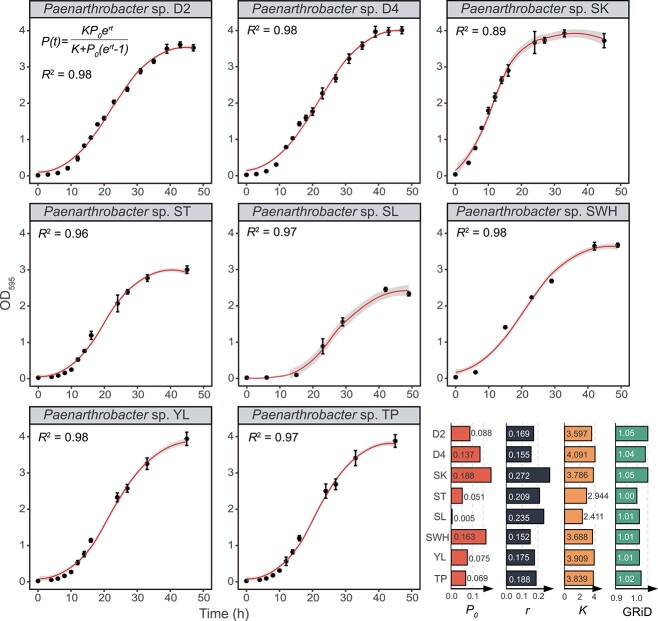
Growth curves of eight *Paenarthrobacter* strains. The growth data was fitted with a logistic model, where *P_0_* indicates the initial population, *r* defines the growth rate, and *K* is the maximum value that the population can reach. The growth rates of isolates under 100-mg/L SDZ pressure were estimated by GRiD (v1.3). Error bars represent the standard deviation from triplicates.

In nutrition-rich conditions, the growth data fitted well with logistic regression (*R*^2^ > 0.89, Fig. 2). *Paenarthrobacter* sp. SK entered exponential growth after inoculation without an obvious lag phase and reached equilibrium after just 24 hours, implying a rapid growth rate of SK and an ability to survive and thrive in the microbial jungle. Meanwhile, the species with a fast growth rate may introduce some unique mutations, thereby enabling a rapid adaption to particular niches [40]. The other seven strains exponentially grew after an approximate 10-hour lag phase before asymptotically reaching equilibrium. Among them, SL and ST achieved lower saturation concentrations (*K*), but the maximum population size (carrying capacity) is independent of the initial value (*P_0_*). To reveal the growth-dependent behavioral difference between pre- and post-antibiotic, we estimated the GRiD of those strains that grew in the medium containing sulfonamide using a sequencing-based approach. Under sulfonamide pressure, SK, D2, and D4 have higher average growth rates (GRiD values) compared to the other five strains, generally agreeing with the result from cultivation in pre-antibiotic conditions. However, in a fluctuating environment, the advantages of short lag time and fast growth rate might average out [41], resulting in a coexistence of diverse sulfonamide-degrading strains in bacterial competition.

### *sad*-dependent degradation performance of *Paenarthrobacter* spp. with strain-level diversity

**Figure 3:**
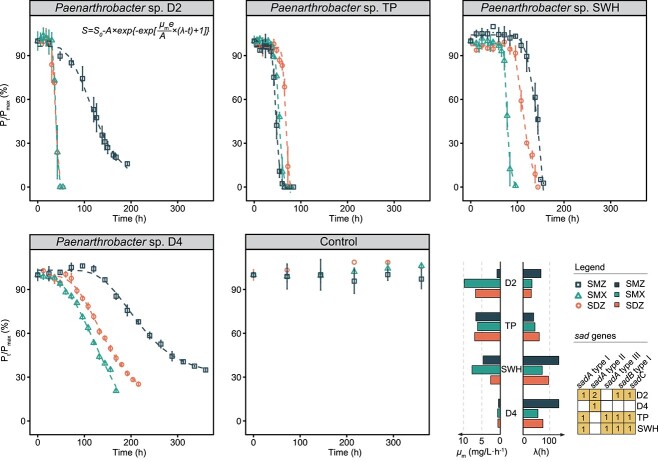
Sulfonamide degradation curves of four selected *Paenarthrobacter* strains in MSM with 100 mg/L sulfonamide (SDZ, SMX, SMZ) under aerobic conditions. All experiments were conducted in triplicate at an initial OD_595_ of 0.1. The degradation data were described using a modified Gompertz model, where *μ_m_* represents the maximum biodegradation rate, and *λ* indicates the lag phase time.

In the present study, four strains were selected for further degradation assay based on their composition of *sad* genes (Fig. S3B). Previous investigations have demonstrated that abiotic processes (e.g. sorption, hydrolysis, and volatility) play a minor role in sulfonamide elimination [42, 43], which is attributed to biotransformation to a large extent (Fig. 3). Herein, all four selected strains exhibited a capability of sulfonamide utilization, and the degradation data could be described well by the modified Gompertz model. Nevertheless, it is inadequate to only focus on parent compound removal, the metabolite generation and bioactivity removal are important as well. Judging from the low total organic carbon removal rate (data not shown), the degree of sulfonamide mineralization by *Paenarthrobacter* was low, which is consistent with the previous study reporting that a low fraction of ^14^CO_2_ could be recovered in a mass balance analysis [44]. Hence, the corresponding dead-end metabolites of sulfonamides were monitored subsequently. The accumulation of three major metabolites (2-aminopyrimidine for SDZ, 3-amino-5-methylisoxazole for SMX, and 4,6-dimethylpyrimidine-2-amine for SMZ) was observed synchronously (Fig. S4), indicating that the degradation was *via* the enzymatic reactions encoded by *sad* genes. In line with previous findings [37, 42, 45], SMX and SDZ were more easily degraded compared with SMZ (Fig. S5). The different heterocyclic moieties (five-member heterocyclic ring for SMX, different six-member heterocyclic rings for SMZ and SDZ) might be responsible for the distinct degradation behaviors [37, 45, 46]. The large-size heterocyclic structure, such as methyl groups on the six-member ring of SMZ, hinders sulfonamide binding to monooxygenase.

In detail, a similar degradation performance was observed between TP and SWH because they have an identical *sad* gene arrangement, namely, two *sadA* (Types I and III), two *sadB* (Types I and II), and one *sadC*. Nevertheless, SWH has the longest lag phase time for all sulfonamides (133.9, 71.2, and 94.5 hours for SMZ, SMX, and SDZ, respectively), probably owing to its relatively low growth rate (Fig. 2). It suggests a longer time is required for SWH to adapt its metabolism to the new environment compared with other strains. Meanwhile, a comparable long lag phase was also observed in D4, but D4 has relatively poor sulfonamide-degrading performance with incomplete parent compound removal due to the possession of only one Type II *sadA* transposable element and a plasmid-borne Type II *sadB*. However, parent compound biotransformation and the release of the major metabolite are only related to the enzymatic reaction involving *sadA* and *sadC* genes (Fig. S1). Moreover, flavin reductase encoded by *sadC* is not specific to the sulfonamide-degrading reaction [18], and therefore, a remotely located flavin reductase–coding gene could also participate in sulfonamide elimination as an electron transporter. In comparison, D2 possesses the most copies of *sadA* genes, including one Type I *sadA* gene (*sad* cluster) and two Type II *sadA* genes (*sadA* transposable element), achieving the best performance for SMX and SDZ biodegradation. D2 could utilize 100 mg/L SMX and SDZ completely with high biodegradation rates (9.8 and 6.7 mg/L^-1^·h^-1^) and short lag phases (33.2 and 30.7 hours). Also, TP could degrade sulfonamides efficiently within 72 hours and reach comparative *μ_max_* values for all three sulfonamides (i.e. 6.2, 6.9, and 6.6 mg/L^-1^·h^-1^ for SMX, SDZ, and SMZ, respectively). In addition, we found that both Type II and III *sadA* can contribute to SMZ elimination, whereas Type II *sadA* could only realize a limited SMZ consumption, even though D2 contains two Type II *sadA* genes. It implied that Type III *sadA* is the key driver of SMZ biodegradation.

### Database mining of *sad* genes

**Figure 4:**
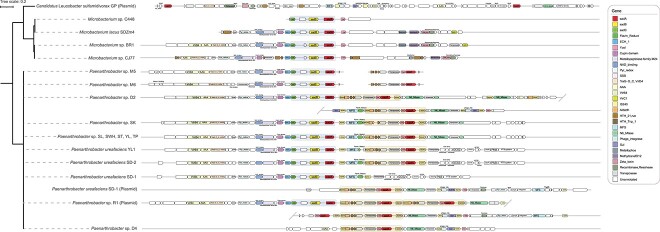
Diverse gene arrangement of sulfonamide-degrading genes in *Microbacteriaceae* spp. and *Micrococcaceae* spp. The phylogenetic tree was rooted using midpoint rooting. *Arthrobacter* spp. M5 and M6 were reassigned to *Paenarthrobacter* by GTDB-tk (v2.1.1) [31] based on Genome Taxonomy Database taxonomy R214.

Unlike natural antibiotics, sulfonamide antibiotics are produced *via* chemical synthesis, and the discovery of sulfonamide-degrading strains is relatively recent. The genes responsible for sulfonamide biodegradation are clustered, comprising two monooxygenases and one flavin reductase. The *sad* cluster originated from *Microbacterium* spp. on the chromosome (Fig. 4) [18, 47-49]. The cleavage of the S-N bond not only allowed the bacteria to utilize the benzene ring as a substrate but also destroyed the antibacterial activity, conferring the ability to survive in environments with sulfonamides. Then, the *sadA* gene, responsible for the first-step cleavage, was integrated into a composite transposon in the later discovered strains, enabling a potential rapid propagation of the target gene within genomes [50-52]. The *sadA*-carrying composite transposon on the chromosome was first reported in *Paenarthrobacter* spp. D2 and D4 (previously named *Arthrobacter*) in 2016 [25]. Subsequently, the convergence of advances in long-read sequencing makes it possible to complete the chromosome and resolve the arrangement of *sad* genes. Based on circular chromosomes, a similar gene arrangement, namely, one *sad* cluster and one *sadA* transposable element, was observed in six newly isolated *Paenarthrobacter* spp. and *Paenarthrobacter ureafaciens* YL1, obtained from activated sludge in 2020, which showed comparable sulfonamide degradation rates [26, 53]. The latest isolated SMX degrader, *Paenarthrobacter ureafaciens* SD-1 [54], revealed a *sadA* gene jumping from chromosome to plasmid, realizing an independent replication of the functional genes. Meanwhile, aggressive insertions of *sadA* were recently reported on a plasmid of *Paenarthrobacter* sp. R1 [37], including four *sadA* transposable elements and one *sad* gene cluster. The densely nested *sadA* transposable elements in *Paenarthrobacter* sp. R1 achieved an efficient utilization of sulfonamides, showing complete removal of 50 mg/L SDZ and SMX within 24 hours. Moreover, it indicated that the cleavage of the S-N bond is the rate-limiting step of sulfonamide biodegradation and multiple *sadA* genes could improve the degradation and inactivation performance. Those observations provided valuable insights into the construction of engineered strains as well as an early warning for the rapid evolution and potentially widespread. Currently, in strains with a complete genome, *sad* cluster and *sadA* transposable element were only identified on nonmobilizable plasmids (CP127116.1 in R1 and NZ_CP101187.1 in SD-1), which was conducive to independent replication but hardly processed horizontal gene transfer. It partially explained that the specialized capability for sulfonamide biodegradation was evolutionarily conserved in three genera, namely *Paenarthrobacter* (previously affiliated with *Arthrobacter*), *Microbacterium*, and *Leucobacter*, and not yet widespread. Additionally, *sad* cluster and *sadA* transposable element were only found in two genera except for *Leucobacter* (*Candidatus* Leucobacter sulfamidivorax GP). However, environments with high concentrations of sulfonamides, such as sulfonamide-degrading enrichments in lab-scale investigations, could facilitate the evolution of antibiotic-resistant microbes, posing a potential threat to ecosystems.

Apart from sulfonamide degraders, homologous genes of Type I *sadB* were identified in three MAGs (Table S5), namely *Nocardioidaceae* bacterium HKST-UBA51 (GCA_020438665.1, 85.0% identity) [38], uncultured *Propionibacterium* sp. SRR8859111_bin.135_CONCOCT_v1.1_MAG (GCA_937867175.1, 83.5% identity), and uncultured *Micropruina* sp. ERR3519522_bin.78_CONCOCT_v1.1_MAG (GCA_937867535.1, 70.2% identity) [55]. They were affiliated with *Propionibacteriaceae* and *Nocardioidaceae* within *Propionibacteriales* and were retrieved from freshwater sediment [56] and wastewater [57] samples, respectively. The *sadB* orthologous genes in *Nocardioidaceae* bacterium and uncultured *Propionibacterium* sp. shared 92.0% identity and were a paraphyletic sister to *sadB* Type I (Fig. S6).

Despite the fact that either *sul* or *sad* gene can confer the bacteria with sulfonamide resistance, the *sul* gene was identified in all *sad*-carrying sulfonamide degraders, which is consistent with a recent study proposing the co-occurrence of *sad* and *sul* genes as a coherent feature of efficient sulfonamide degraders [54]. However, even though *Paenarthrobacter* sp. SK contained a complete *sad* gene cluster, *sadA* transposable element, *sul918*, and *sul1*, it displayed a limited ability to utilize sulfonamide as the sole carbon or energy source [26]. This result was not fully in agreement with the conclusion that the lack of *sul918* leads to the poor SMX-degrading performance of *Paenarthrobacter ureafaciens* SD-2, which contains a complete *sad* gene cluster, *sadA* transposable element, and *sul1* gene [54]. Compared with other strains, the *sadA* transposable element of SK is incomplete (Fig. 4). The lack of the transposase may result in the low expression of nearby genes [58, 59], but more solid evidence is still needed to confirm this suspect. Generally, the current results were in accordance with the notion that the *sul* genes contributed to sulfonamide resistance, whereas *sad* genes were responsible for sulfonamide degradation, and the combination of *sul* and *sad* genes enhanced the biodegradation performance.

In the present study, we only monitored the parent compound degradation and the formation of corresponding dead-end metabolites, but mass balance analysis is still needed to resolve quantitative differences in sulfonamide-degrading performance among *Paenarthrobacter* strains. Moreover, transcriptomic analysis could be further applied to verify the contribution of Type III *sadA* to SMZ degradation and the involvement of Type II *sadB* in sulfonamide biodegradation. On the other hand, pure culture results generally cannot be extrapolated to mixed communities, and serial issues on biosafety of these rapid evolution strains remain to be addressed before practical application. Lastly, antibiotic use is a double-edged sword for bacteria, which kills them but also facilitates the evolution of resistance genes as well as degradation genes, reminding the relationship between nature and humans.

## Conclusions

In light of the widespread use of sulfonamides, the evolutionary pressure for the emergence of antibiotic resistance is great. Under this circumstance, biodegradation plays a crucial role in sulfonamide elimination in natural environments. In the present study, we explored the strain-level differentiation in *Paenarthrobacter* on sulfonamide biodegradation. Our results revealed that SDZ and SMX were easy to degrade, whereas SMZ was relatively recalcitrant probably owing to its heterocyclic ring. The sulfonamide-degrading performance was generally related to *sadA* numbers, but SMZ biodegradation might rely on the *sadA* genotype. Based on thesurvey of *sad* genes in the public database, *sad* gene cluster and *sadA* transposable element were conserved in two genera (i.e. *Paenarthrobacter* [*Micrococcaceae*] and *Microbacterium* [*Microbacteriaceae*]). In addition, *Paenarthrobacter* demonstrated a high capacity for genome editing, propagating multiple *sadA* genes in both chromosome and plasmid and promoting sulfonamide biodegradation, which opened up new possibilities for engineering applications.

## Supplementary Material

SMs_MS_SI_v4_wrad040

SI_table_v4_wrad040

## Data Availability

The raw data of short- and long-read sequences have been deposited in the NCBI database under project ID PRJNA669352. The reassembled genomes were deposited under BioSample Accession numbers SAMN38477253–SAMN38477260. Raw data of additional long-read sequences are available from the corresponding author on reasonable request.
