# Supplementary material for: Strain-level diversity in sulfonamide biodegradation: adaptation of Paenarthrobacter to sulfonamides
Source: ISME J. 2024 Jan 10;18(1):wrad040. doi: 10.1093/ismejo/wrad040 (PMC10873849; doi:10.1093/ismejo/wrad040)
Supplement: SMs_MS_SI_v4_wrad040 [file sms_ms_si_v4_wrad040.docx]

**Supplementary Materials for**

**Strain-level diversity in sulfonamide biodegradation: adaptation of *Paenarthrobacter* to sulfonamides**

Yue Huang, Anxin Pan, Ying Song, Yu Deng, Alnwick Long-Hei Wu, Colin Shiu-Hay Lau, Tong Zhang^*^

Environmental Microbiome Engineering and Biotechnology Lab, Department of Civil Engineering, The University of Hong Kong, Pokfulam Road, Hong Kong, China.

*Corresponding author.

Mailing address: Room 6-31, Haking Wong Building, Department of Civil Engineering, The University of Hong Kong, Pokfulam Road, Hong Kong SAR 999077, China. Email address: zhangt@hku.hk (T. Zhang).

**List of Figures**

Figure S1. Degradation pathways of sulfonamides catalyzed by the *sad* genes.

Figure S2. Phylogenetic tree of the eight isolates and other *Arthrobacter* and *Paenarthrobacter*.

Figure S3: Genome comparison of *Paenarthrobacter* genomes and *sad* gene clusters in four strains used in the degradation assay.

Figure S4. Biodegradation of sulfonamides and generation of corresponding metabolites by different strains

Figure S5. Sulfonamide degradation curves of four selected *Paenarthrobacter* strains.

Figure S6. Maximum likelihood tree of *sadB* homologous genes based on amino acid sequences.

**List of Tables (Supplementary Table)**

Table S1. The composition of mineral salt medium (MSM).

Table S2. Summary of transformation products identified in sucralose biodegradation samples using UPLC−MS/MS.

Table S3. The genomic information of eight isolates.

Table S4. Pairwise ANI and AAI values among the strains.

Table S5. Summary of the genome-centric survey of *sad* genes.

Figure S1. Degradation pathway of sulfamethoxazole (SMX), Sulfadiazine (SDZ), and sulfamethazine (SMZ) catalyzed by the *sad* genes. This figure is modified from Figure 5 in the previous study [1]. TP99: 3-amino-5-methylisoxazole; TP96: 2-aminopyrimidine; TP124: 2‑amino‑4,6‑dimethylpyrimidine.


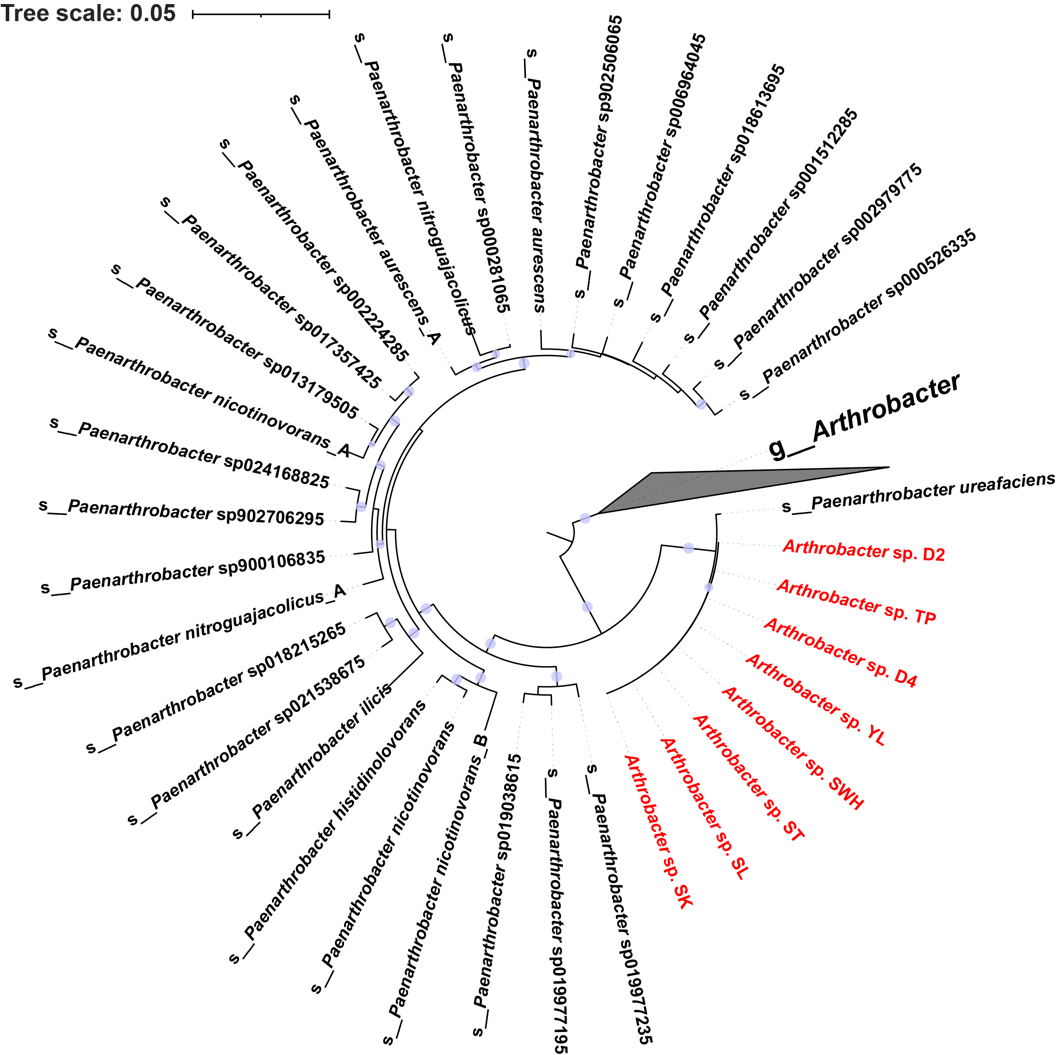


Figure S2. Phylogenetic tree of the eight isolates and other *Arthrobacter* and *Paenarthrobacter* with publicly available based on the genome phylogeny. Only bootstrap values >70% are shown.


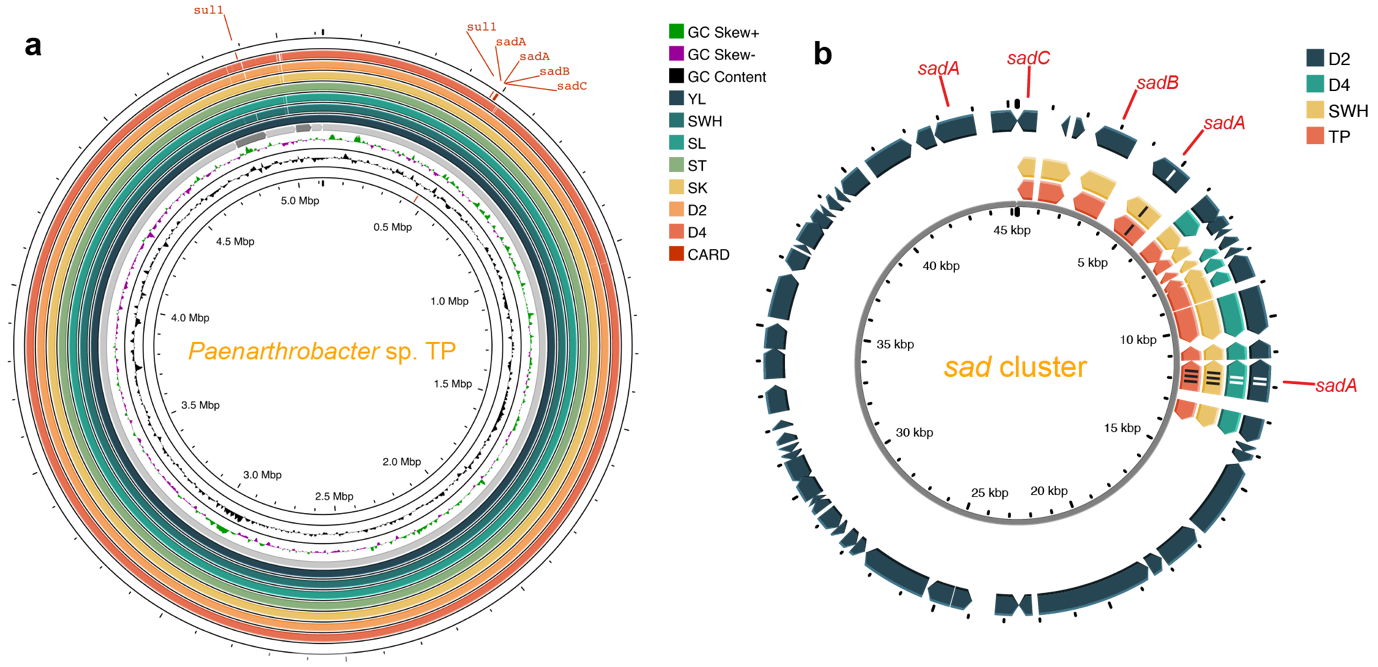


Figure S3: (a) Genome comparison of *Paenarthrobacter* genomes. Graphical circular maps were generated with CGview [2]. (b) *sad* gene clusters of four strains used in the degradation assay.

Figure S4. (a) Biodegradation of sulfadiazine (SDZ), sulfamethoxazole (SMX), and sulfamethazine (SMZ) by different strains. (b) The simultaneous accumulation of major metabolites, 2-aminopyrimidine, 3-amino-5-methylisoxazole, and 4,6-dimethylpyrimidin-2-amine, respectively.

Figure S5. Sulfonamide degradation curves of four selected *Paenarthrobacter* strains. The degradation data was described using a modified Gompertz model, where *μ_m_* represents the maximum biodegradation rate, and *λ* indicates the lag phase time.


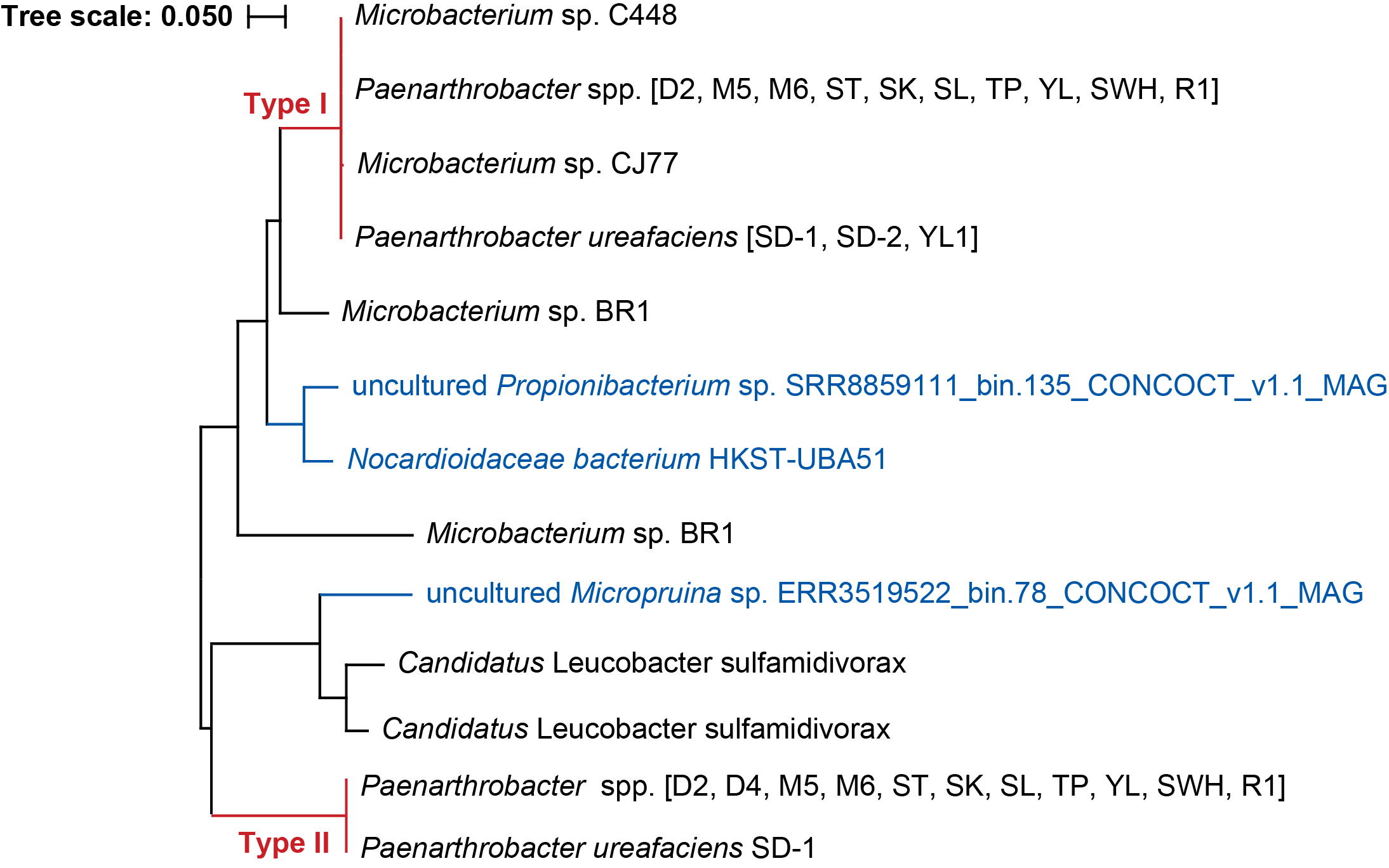


Figure S6. Maximum likelihood tree of *sadB* homologous genes based on amino acid sequences. In the genome-centric survey, The open reading frames were predicted by Prodigal, and homologous genes were identified using DIAMOND with strict criteria. The strains in blue fond were *sadB*-carrying metagenome-assembled genomes retrieved from wastewater and freshwater sediment samples. Either the *sadA* or *sadC* was identified in them.

**Reference:**

1. Ricken B, Kolvenbach BA, Bergesch C, Benndorf D, Kroll K, Strnad H *et al* (2017). FMNH2-dependent monooxygenases initiate catabolism of sulfonamides in *Microbacterium* sp. strain BR1 subsisting on sulfonamide antibiotics. Sci Rep 7**:** 15783.

2. Grant JR, Stothard P (2008). The CGView Server: a comparative genomics tool for circular genomes. Nucleic Acids Res 36**:** W181-W184.
